# The Influence of Hyaluronic Acid Adjunctive Therapy of Periodontitis on Salivary Markers of Oxidative Stress: Randomized, Controlled Clinical Trial

**DOI:** 10.3390/antiox11010135

**Published:** 2022-01-07

**Authors:** Iwona Olszewska-Czyz, Kristina Kralik, Marin Tota, Jelena Prpic

**Affiliations:** 1 Department of Periodontology, Prophylaxis and Oral Pathology, Dental Institute, Medical Faculty, Jagiellonian University, 31155 Krakow, Poland; 2 Department of Medical Statistics and Medical Informatics, Medical Faculty Osijek, University Josip Juraj Strossmayer of Osijek, 31000 Osijek, Croatia; kristina.kralik@gmail.com; 3 Department of Medical Chemistry, Biochemistry and Clinical Chemistry, Medical Faculty, University of Rijeka, 51000 Rijeka, Croatia; marin.tota@medri.uniri.hr; 4 Department of Oral Medicine and Periodontology, Faculty of Dental Medicine, University of Rijeka, 51000 Rijeka, Croatia; jelena.horvat.prpic@gmail.com

**Keywords:** antioxidant status, oxidative stress, periodontitis

## Abstract

Periodontitis is a common oral disease affecting the tooth-supporting tissues. Bacteria have been long viewed as the main causative factor in its development; however, many investigations have proved that aberrant immune and inflammatory response and the resulting misbalance between the damage caused by reactive oxygen species and the antioxidant capacity of tissues may be an underlying factor in disease progression that reduces healing potential. The objective of the current trial is to assess the outcomes of the addition of hyaluronic acid (HA) to standard non-surgical periodontal therapy (NST) on some major oxidative stress markers in saliva. HA-based gel designed for dental application was used and the measurements were taken after 3 months. HA adjunctive therapy had a significantly greater increase in markers with antioxidant properties as well as total antioxidant capacity compared to standard NST alone. Furthermore, clinically measured levels of gingival inflammation (bleeding on probing-BOP) and periodontal destruction (clinical attachment loss-CAL) were significantly correlated with these markers, and the correlation was negative. This investigation demonstrates that HA may indeed express antioxidant properties and improve the antioxidant capacity of periodontal tissues, thus improving the prognosis for the teeth and the results of periodontal therapy. Further investigations will be necessary to determine the duration of these effects over time.

## 1. Introduction

Chronic diseases have followed humankind for thousands of years, knowing no boundaries or socioeconomic status and showing no sign of relenting even in the so-called “first-world countries”. Medical advances and healthy lifestyles have certainly helped keep the levels of undesirable outcomes of these diseases under control; however, we have not been able to eliminate them completely. Periodontitis is one of the most common components of the global burden posed by chronic diseases, and its development is strongly affected by both inherent and environmental factors [1]. Bacterial dysbiosis is considered the main causative factor that, in genetically susceptible individuals, triggers a strong inflammatory and immune response. This is further aggravated by diseases such as diabetes or obesity, habits such as smoking, and—common in the modern-day world—stress [2,3]. The above-mentioned inflammatory and immune host responses play a crucial role in disease progression, especially in cases that do not respond well to therapy. In this context, many authors have suggested the role of reactive oxygen species and subsequent oxidative stress. It is stipulated that these play a significant role in the pathogenesis of not only periodontitis but also other chronic inflammatory diseases such as diabetes, atherosclerosis, rheumatoid arthritis, cancer, and inflammatory lung disease [4,5,6,7].

In health, reactive oxygen species’ main role is helping to kill invading pathogenic microorganisms; they are also important in cell signaling, gene regulation, and antimicrobial defense [8]. In the case of misbalance, an overabundance and the resulting excess of their activity, coupled with unaltered or reduced antioxidant capacity, lead to pathological changes and, consequently, the destruction of host tissues. In the pathogenesis of periodontitis, polymorphonuclear neutrophils are viewed as the primary source of this misbalance. As first-responder cells, they act in several ways, including degranulation, chemotaxis, phagocytosis, and the release of reactive oxygen species [9]. During phagocytosis, the free radicals that are subsequently released cause lipid peroxidation but may also damage protein and DNA. This triggers proinflammatory reactions, including osteoclast activation, leading to bone loss as one of the main findings in periodontal lesions [10].

Numerous investigations and reports have demonstrated the reduced antioxidant capacity and increased levels of oxidative stress markers in saliva, gingival crevicular fluid, and plasma of patients with periodontitis [11]. Intervention studies have proved that non-surgical periodontal therapy alone improves the levels of oxidative stress markers and antioxidant capacity [12]. In a research setting, there is a palette of oxidative stress markers, and some interventional studies indicate that antioxidant treatment could have therapeutic value as an addition to the standard non-surgical treatment of periodontitis or might even slow down the progress of periodontitis [11,12]. These studies also raised the question of whether some particular intervention or adjuvants could offer additional benefit in reversing the tissue damage cycle and the recovery of periodontal lesions.

The European Federation of Periodontology (EFP) has recently strengthened the importance of non-surgical periodontal therapy (first and second steps), which consists of professional mechanical plaque removal (PMPR) coupled with patient motivation, modifications in lifestyle, instructions for the optimum maintenance of oral hygiene, and subgingival instrumentation [13]. Second step therapy may include the use of adjunctive physical or chemical agents, host-modulating agents (local or systemic), subgingival locally delivered antimicrobials, or systemic antimicrobials. Among the many reviewed agents that are commonly used in this second-step treatment, the only ones with antioxidant properties were the statin gels. Although the mean estimates suggested a clinically meaningful benefit from adding statin gels to mechanical instrumentation, the panel led by Prof. Nicolaos Donos concluded that they could not recommend the local administration of statin gels (atorvastatin, simvastatin, rosuvastatin) as adjuncts to mechanical instrumentation due to a moderate risk of bias, the wide heterogeneity of obtained data, and the unclear level of involvement of industry in the analysis [14]. Nevertheless, a literature search offers many more antioxidant candidates that may be used as adjuncts to non-surgical mechanical therapy, including vitamins C and E, lycopene, Er:YAG laser, zinc, selenium, coenzyme Q10, tea tree oil, even dietary interventions and—quite surprisingly—tai chi. All of them were viewed as possible tools that could affect not only oxidative damage but also the inflammatory process and interfere with variable cellular signaling pathways [15]. Although the effects and biological activity of hyaluronic acid—including its antioxidant properties—have already been established in numerous in vivo and in vitro investigations and the level of scientific evidence is rather significant [16,17], there are no clinical trials that have assessed the effect of its addition to periodontal therapy on antioxidant capacity and the levels of oxidative stress markers. Therefore, we have decided to test the hypothesis that the use of hyaluronic acid as a local adjunct to non-surgical periodontal therapy will improve the levels of oxidative stress markers and the overall antioxidant capacity in healthy, non-smoking patients diagnosed with periodontitis.

Hyaluronic acid gel applied in the current trial is defined in composition and properties (Hyadent BG^®^, BioScience GmbH, Dummer, Germany). It is a mix of 16 mg/mL cross-linked HA and 2 mg/mL non-cross-linked HA with the molecular weight of 1 million Dalton and it is obtained by bacterial fermentation (*Streptococcus zooepidemicus*). The cross-linking degree is between 0% and 20%, and the cross-linking process includes using BDDE (1,4-butanediol diglycidyl ether) on alkaline pH.

The aim of the study is to evaluate the effect of non-surgical therapy with the addition of hyaluronic acid in cases of moderate periodontitis on oxidative stress markers in saliva. The main research objective is to investigate if the use of hyaluronic acid in periodontal therapy has any additional effect on the antioxidant potential and to analyze possible differences in levels of oxidative stress markers with or without the local application of HA.

## 2. Materials and Methods

### 2.1. Trial Design

**Figure 1:**
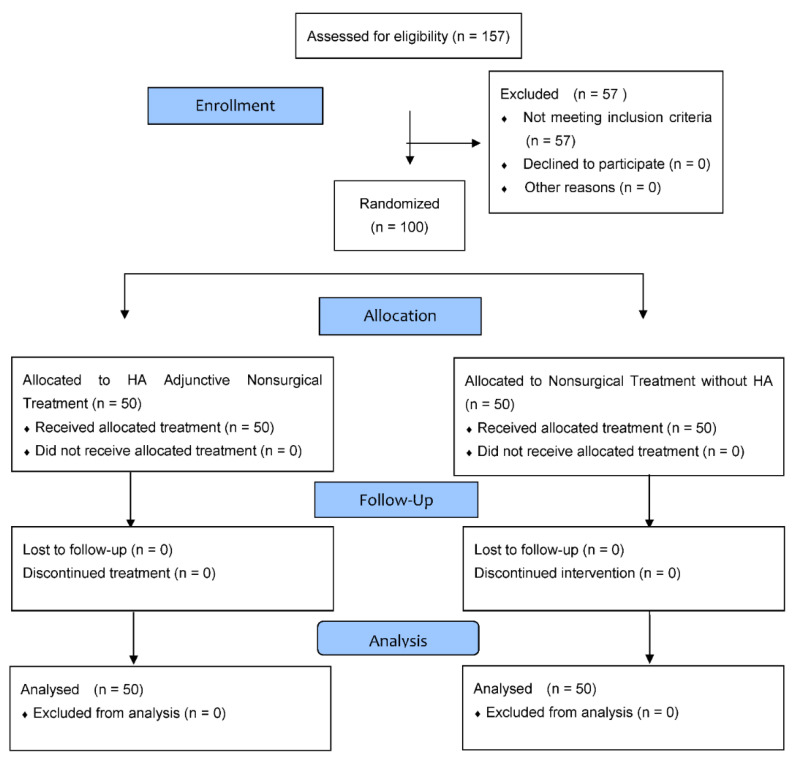
CONSORT 2010 flow diagram.

The duration of the trial was 3 months. It was a single-center, prospective, randomized, controlled, single-blinded investigation. Patients from the Periodontal Division of the University Dental Clinic in Cracow (Poland) were enrolled. The Helsinki Declaration was applied, and all the participants gave written consent to participate. Official approval was obtained (Jagiellonian University Ethics Committee, No. 122.6120.132.2015). The enrollment took place during scheduled appointments. The study design follows CONSORT guidelines (Figure 1).

### 2.2. Randomization and Blinding

Study participants received code numbers, and a randomizing software was used to allocate patients to one of the two groups (non-surgical treatment only study group and non-surgical treatment with adjunctive hyaluronic acid (HA) treatment control group) (allocation ratio: 1:1, Research Randomizer Computer Software Version 4.0 from http://www.randomizer.org, accessed on the 9 October 2020) [18]. Participants were blinded as the hyaluronic acid was applied by an anesthetic syringe, which was also used in both study groups for anesthesia.

### 2.3. Participants

One hundred adult participants (51% female, aged from 25 to 65 years) were enrolled. They were generally healthy and recruited after completing the first step of the periodontal therapy no later than 4 weeks before the study. They had to present an Approximal Plaque Index (API) lower than 25%, and they had to demonstrate localized moderate periodontitis with a minimum of two sites with a periodontal probing depth (PPD) of 4 mm or more. The diagnosis was confirmed by radiographs and was based on the examination in accordance with the 2017 World Workshop on the Classification of Periodontal and Peri-Implant Disease and Conditions [19,20]. Exclusion criteria were as follows: antibiotic therapy within the last 6 months; non-steroid anti-inflammatory drugs, corticosteroids, or multivitamin supplements within the last 3 months; smoking (last 5 years); caries; epithelial dysplasia; any inflammatory lesions of the oral mucosa; rheumatic disorders; Sjögren disorder; enteritis; asthma; sinusitis; pregnancy; and receiving periodontal treatment within the last 6 months before the trial.

### 2.4. Data Collection

Data collection took place at the baseline and after 12 weeks. Medical history, medication use, demographics, and oral hygiene routine were recorded, and the clinical parameters were measured. Unstimulated saliva samples (4 mL) were collected in sterile plastic tubes.

### 2.5. Clinical Parameters

A single periodontal examiner performed the following oral examination: Approximal Plaque Index (API) [21], bleeding on probing (BoP) [22], periodontal probing depth (PPD), and clinical attachment level (CAL). The instrument used was a periodontal probe (PCP-UNC 15, Hu-Friedy, Chicago, IL, USA).

### 2.6. Intervention

The hyaluronic acid (HA) adjunctive treatment study group (*n* = 50) received non-surgical periodontal therapy, including subgingival instrumentation, followed by HA application to the existing pockets, while the control group (*n* = 50) received only non-surgical periodontal therapy without HA application [23]. The non-surgical one-session instrumentation (full mouth debridement) for both groups was applied at the baseline and after six weeks. HA-based gel was applied as adjunctive to the existing pockets in the study group at both times. Hand curettes were used (Hu-Friedy, Chicago, IL, USA). Patients were followed up after 12 weeks. Patients were referred for follow-up periodontal care after the trial. Consecutive supportive periodontal therapy was provided.

### 2.7. Saliva Preparation for the Oxidant–Antioxidant Tests

Unstimulated saliva samples (4 mL) were collected in sterile plastic tubes using the Salivette^®^ Cotton Swab system (Sarstedt, Nuembrecht, Germany). The biological material was collected in the morning, between 9:00 and 11:00 AM. The samples were taken before any treatment was undertaken. The subjects rinsed their mouths with tap water for 30 s and expectorated it before the saliva was collected. The study subjects did not eat, drink, brush their teeth or chew gum for a minimum of 2 h prior to the sampling. The saliva was centrifuged at 900× *g* for 10 min at a temperature of 4 °C. Then, the entire filtrate was transferred to sterile 1.5 mL micro test tubes (Eppendorf type) and frozen at −80 °C until analysis.

### 2.8. Measurement of Oxidative Stress Parameters

Four oxidative stress parameters—total antioxidant capacity (TAC), concentration of uric acid (UA), concentration of total glutathione (GSH), and activity of glutathione reductase (GR)—were measured in the saliva.

#### 2.8.1. Total Antioxidant Capacity (TAC)

Total antioxidant capacity was determined by the ferric reducing/antioxidant power (FRAP) assay provided by Benzie et al. [24]. Low molecular weight antioxidants from the sample reduced the Fe(III) ions present in the complex with tripyridyl triazine (TPTZ) as Fe(III)–TPTZ to Fe(II)–TPTZ under acidic conditions. The resulting Fe(II)–TPTZ complex was characterized by intense blue color and had a maximum absorption at wavelength λ = 593 nm. Acetate buffer (pH = 3.6), TPTZ solution, and FeCl_3_ • 6H_2_O were mixed in a 10:1:1 ratio to obtain a working FRAP solution. For the calibration of the FRAP assay, the 100 mM FeSO_4_ solution was used and diluted in a range from 0 to 2.5 mM. The sample’s antioxidant capacity was determined by comparing the changes in absorbance ΔA with the value of ΔA of the standard solution Fe(II). The final result was expressed in the form of mmol/l, using a Biotek ELX808 microplate reader. All reagents used in the assay were from Sigma Aldrich (St. Louis, MO, USA). The intra-assay and inter-assay precisions were 3.2% and 7.0%, respectively.

#### 2.8.2. Total Glutathione (GSH)

The concentration of total glutathione (the sum of GSH and GSSG) was assessed by the use of an OxiSelectTM Total Glutathione (GSSG/GSH) Assay Kit (Cell Biolabs, INC, San Diego, CA, USA) and an EL-800 automatic microplate reader (Bio-Tek Instruments, INC, Winooski, VT 05404, USA). The method is based on the formation of a color product in the tested material. Measurement of the resulting color was made at wavelength λ = 405 nm [25]. Glutathione concentration refers to a standard curve prepared from a GSSG standard solution, and it is expressed in µmol/l. The final values are expressed in µmol/mg of total saliva protein. The protein concentrations of the samples were determined on the basis of the bicinchoninic acid (BCA) method in accordance with the manufacturer’s (Sigma-Aldrich, St. Louis, MO 63118, USA) instructions, with bovine serum albumin (BSA) adopted as the standard.

#### 2.8.3. Glutathione Reductase Activity (GR)

Glutathione reductase activity was determined on the basis of the calculation of glutathione-reductase-catalyzed oxidation reduction of glutathione (GSSG) in the presence of NADPH, which undergoes oxidation and conversion to NADP [26]. The absorbance decrease for NADP was measured at wavelength λ = 340 (Goldberg, 1983). The final result is expressed in U/g of the total saliva protein.

#### 2.8.4. Uric Acid Concentration (UA)

The uric acid concentration was assessed on the base of the uricase (enzymatic-colorimetric) method of uric acid oxidation to allantoin and hydrogen peroxide [27]. The hydrogen peroxide reacts with peroxidase and 4-aminoanthytirine and *N*-ethyl-*N*-(2-hydroxy-3-sulfopropyl)-m-toluidine (EHSPT), creating a colored product, which was measured at 550 nm wavelength. The amount of the dye is proportional to the amount of uric acid present in the sample. The final result is expressed in µmol/L.

### 2.9. Safety Monitoring

Oral symptoms were recorded at the baseline, after six weeks, and after twelve weeks. During the follow-up examination, the patients were questioned if they had experienced any diverse events.

### 2.10. Statistical Analysis

Data were described using descriptive statistical methods. The Mann–Whitney U-test and the Wilcoxon test were used to compare the median between two groups or two measurements (with 95% CI). Spearman’s rho test was used to determine the association between continuous variables. Alpha = 0.05 was set as a level of significance. The calculated minimum required sample size was 44 subjects per group (the effect size 0.5, level of significance 0.05, power 0.9). Statistical analysis was performed using MedCalc^®^ Statistical Software version 19.6 (MedCalc Software Ltd., Ostend, Belgium (https://www.medcalc.org; accessed on the 9 May 2020) and SPSS (IBM Corp. Released 2013. IBM SPSS, Ver. 21.0. Armonk, NY, USA).

### 2.11. Adverse Events and Safety Monitoring

There were no adverse events reported in any of the patients throughout the study. The regimen was completed by all participants.

## 3. Results

One hundred participants took part in the trial (median age 51 years in the control group and 52 years in the study group). No significant differences were observed for the measured parameters regarding gender; additionally, the age of patients had no significant effect on oxidative stress parameters before or after therapy. At baseline, no significant differences were observed between the two groups for all tested parameters. After the therapy, patients with the addition of HA had significantly higher values of antioxidant capacity (Mann–Whitney U-test, *p* = 0.02) and concentrations of uric acid (Mann–Whitney U-test, *p* < 0.001) compared to standard periodontal treatment only (Table 1).

Both groups showed significant differences in all observed parameters before and after therapy (Wilcoxon test, *p* < 0.001) (Table 2), i.e., the overall antioxidant capacity was improved by both treatment protocols.

The improvement in oxidative stress parameters was more pronounced in the group with HA; the differences were statistically significant for TAC, UA, and GSH (Table 3).

Patients with standard therapy (no HA) presented a statistically significant negative correlation between BoP and CAL values before therapy and oxidative stress parameters. As for the follow-up after therapy, only the BoP values after therapy were significantly correlated (again, the correlation was negative) with oxidative stress parameters, both before and after therapy (Table 4).

In patients with HA, BoP and CAL before therapy correlated negatively with all oxidative stress parameters before therapy, while PPD values before therapy correlated negatively with TAC and GR. Following therapy, BoP correlated significantly (the correlation was negative) with all oxidative stress parameters before therapy and TAC, GR, and UA values after therapy; mean CAL after therapy correlated negatively with all oxidative stress parameters before therapy, but not after therapy, while PPD values after the therapy correlated negatively with TAC and GR values before therapy and all parameters of oxidative stress after therapy (Table 5).

## 4. Discussion

Oxidative stress should not be simply viewed as a part of a simple mathematical equation where the excess of reactive oxygen or nitrogen species in the conditions of reduced antioxidant capacity leads to tissue damage but also as an important physiological process that enables the immune system to cope with microorganisms and intracellular cell signaling. The opposing conditions that can thus be observed are oxidative distress versus oxidative eustress [28]. The role of oxidative stress in periodontitis was first analyzed many years ago [29,30], and observational studies analyzing oxidative stress in patients with periodontitis had markedly consistent results showing higher gingival crevicular fluid/salivary/blood levels of oxidative stress markers and/or decreased antioxidant status compared to controls. Unlike the observational studies, which are quite numerous (more than 50 published in the English language), interventional studies are significantly fewer and cover many different interventions that were not always limited or even related to periodontal therapy [31]. In this interventional study performed on 100 patients, we show that both non-surgical periodontal therapy and non-surgical periodontal therapy coupled with the addition of hyaluronic acid gel significantly increase the levels of markers with antioxidant properties (glutathione reductase—GR, uric acid—UA, total glutathione concentration—TGC) and total antioxidant capacity (TAC) in the saliva of the patients after three months of the follow-up period.

Saliva is an easily obtained diagnostic tool that contains various markers of oxidative stress produced locally. Previously published investigations have proved that it is equally valid as blood and gingival crevicular fluid and the results obtained are comparable; this is very important from a methodological standpoint since it enables the application of non-invasive tools (especially saliva collection) [32]. The above-mentioned oxidative stress markers can be grouped into three categories: lipid peroxidation, protein oxidation, and DNA oxidation markers. Finally, there is the antioxidant capacity/status as the least specific category. Total glutathione (GSH), glutathione reductase (GR), and uric acid (UA) are some of the most common enzymatic antioxidants: GSH is the most abundant intracellular antioxidant, which is also often referred to as the body’s master antioxidant; GR catalyzes the reduction of glutathione disulfide (GSSG) to the sulfhydryl form glutathione (GSH), and uric acid is responsible for more than a half of the antioxidant capacity in human blood [33].

Non-surgical periodontal therapy (NSPT) is the treatment of choice as phase 1 in cases of periodontitis, as recommended by EFP S3 level clinical practice guidelines [23]. Its primary aim is to reduce the overall microbial burden and reduce inflammation of periodontal tissues; however, many published randomized controlled investigations have also shown an improvement of oxidative stress parameters and/or antioxidative status with or without a particular adjuvant [34,35,36,37,38]. Abou Sulaiman and Shehadeh [34] showed that NSPT was effective in improving the antioxidant status at one-month follow-up but with no significant additional effect of vitamin C. This study was performed on only 30 patients and measured the total antioxidant capacity. Two studies proved that the addition of lycopene gel [35] and vitamin E [36] significantly reduced the levels of oxidative injury markers; however, no such effect was demonstrated for dietary counseling [37] or Er:YAG laser [38]. Lycopene gel was evaluated on only 31 patients and vitamin E on 60 patients in total, with follow-up periods of six and three months, respectively. The number of subjects was, again, quite low with Er:YAG laser (30 patients) and dietary counseling (51 patients in total), with follow-up periods of three and six months. Adequate comparison of the available study results is very difficult for many reasons: use of different oxidative stress markers (superoxide dismutase, 8-hydroxydeoxyguanosine) and markers for antioxidant status (most commonly used is total antioxidant capacity; huge heterogeneity in treatment protocols and adjuvants; lack of patient selection criteria, depending on their smoking and diabetes status; many studies were not randomized, lacked control group, lacked power due to a small number of patients, had short follow-up periods, or were undertaken on animals [39]. Finally, there are no available studies assessing the oxidative stress parameters or antioxidant status before and after non-surgical periodontal therapy with the addition of hyaluronic acid.

Another challenge in the discussion of our results is the fact that there are no available studies that have analyzed the correlation between periodontal parameters and oxidative stress markers/antioxidant capacity. We found that both treatment groups (regardless of the HA addition) showed a negative correlation between BoP and CAL values before treatment and oxidative stress parameters and TAC both before and after treatment. This means that the higher degree of inflammation and the greater extent of periodontal destruction at baseline (as measured by BoP and CAL) are directly related to lower antioxidant capacity and higher levels of oxidative stress; this correlation remains even after treatment, thus reducing the possibility of adequate tissue recovery. It is well known that in clinical settings, patients with more advanced periodontal disease have a poorer prognosis and, often, inadequate healing and, as such, require either the addition of antibiotics or subsequent surgical treatment, which not only increases the overall cost but is also time-consuming and somewhat traumatic for the patient [40,41]. In this investigation, we demonstrated that the levels of oxidative stress markers were significantly more reduced and levels of total antioxidant capacity were more increased upon the addition of hyaluronic acid as a part of the non-surgical treatment; therefore, this biological macromolecule may be viewed as a potentially important tool/material to improve the chances of periodontal tissue recovery and increase the success of non-surgical periodontal therapy.

Hyaluronic acid has already been proved as an extremely interesting biomolecule with an array of effects, from signaling, space-occupying, lubricating joints, and trapping water inside various tissues to anti-inflammatory and regenerative effects; in the oral cavity, it has been found that it also displays anti-edematous, anti-bacterial, and pro-angiogenetic properties [42]. An investigation by Ke et al. [43] found that hyaluronic acid has pronounced free radical scavenging and antioxidant activities, while Bergandi et al. [44] proved that the loss of hyaluronic acid in the corpus vitreous was directly related to oxidative stress and lipid peroxidation.

The main strength of this investigation is the fact that, to our knowledge, this is the first randomized controlled investigation that has assessed the effect of the local application of hyaluronic acid on oxidative stress and antioxidant status when used as a part of a non-surgical treatment protocol for periodontitis. Another advantage is the patient selection criteria, which excluded smokers and diabetic patients: it has been shown that smoking status and certain diseases such as type 2 diabetes can significantly alter both the baseline values of oxidative stress markers and the response to therapy [45,46]. The main limitation is the selection of oxidative stress markers (they should include both markers of stress and antioxidant capacity, whereas we only analyzed the molecules pertaining to antioxidant status) and a relatively short follow-up period.

Future investigations should undoubtedly include more markers from different categories and extend the follow-up period to 6 and 12 months in order to observe the molecular changes in different time frames. As for the clinical recommendations, it would be very interesting to observe the effect of different HA application protocols on oxidative stress over time. Investigations should also include some novel antioxidants, such as melatonin, which seems most promising at the moment, according to animal experiments [47,48].

## 5. Conclusions

Non-surgical periodontal therapy contributes to the improvement of antioxidant status in the oral cavities of patients suffering from periodontitis. Data obtained from this randomized study indicate that the hyaluronic acid addition to standard non-surgical periodontal therapy further improves this antioxidant status, as measured by the levels of markers of antioxidant properties and total antioxidant capacity. Reduced levels of gingival inflammation and greater periodontal attachment gain were correlated with this improved antioxidant status; therefore, from the practical standpoint, it would be possible to recommend the clinical use of hyaluronic acid as an addition to the standard non-surgical periodontal therapy in everyday settings. However, more randomized controlled trials—especially long-term—are necessary in order to assess whether such favorable effects remain for longer periods of time.

## Figures and Tables

**Table 1:** Values before and after therapy for both groups.

	Standard Therapy	Standard Therapy + HA	Difference ^†^(95% CI)	*p* *
Before therapy				
TAC—total antioxidative capacity	0.79 (0.6–1)	0.77 (0.6–1)	−0.003 (−0.09 to 0.05)	0.94
GR—glutathione reductase	10.3 (7.3–12.3)	9.7 (7.42–12.5)	−0.025 (−0.97 to 0.67)	0.87
UA—uric acid	237 (201–265)	226.5 (196–284.75)	0 (−19 do 16)	0.99
GSH—total glutathione concentration	3.79 (1.6–5)	2.99 (1.55–5.66)	−0.09 (−1.04 to 0.48)	0.73
After therapy				
TAC	0.89 (0.8–1)	0.98 (0.81–1.08)	0.08 (0.01 to 0.13)	0.02
GR	11.11 (8.3–12.8)	10.67 (8.76–12.96)	0.2 (−0.49 to 0.81)	0.47
UA	275.5 (256–296)	296 (286.3–327.8)	26 (14 to 39)	<0.001
GSH	3.92 (2–5.4)	3.86 (2.56–6.01)	0.3 (−0.45 to 0.96)	0.35

HA—hyaluronic acid; 95% CI—95% confidence interval; * Mann–Whitney U-test; ^†^ Hodges–Lehman median difference.

**Table 2:** Values of observed parameters before and after therapy within every group.

	Median (Interquartile Range)		Difference ^†^(95% CI)	*p* *
	Before Therapy	After Therapy		
Standard therapy				
TAC	0.79 (0.6–1)	0.89 (0.8–1)	0.06 (0.03 to 0.09)	<0.001
GR	10.3 (7.3–12.3)	11.11 (8.3–12.8)	0.59 (0.45 to 0.72)	<0.001
UA	237 (201–265)	275.5 (256–296)	36 (27 to 45.5)	<0.001
GSH	3.79 (1.6–5)	3.92 (2–5.4)	0.31 (0.23 to 0.40)	<0.001
Standard therapy + HA				
TAC	0.77 (0.6–1)	0.98 (0.81–1.08)	0.15 (0.12 to 0.19)	<0.001
GR	9.7 (7.42–12.5)	10.67 (8.76–12.96)	0.9 (0.65 to 1.17)	<0.001
UA	226.5 (196–284.75)	296 (286.3–327.8)	64.5 (53.5 to 77)	<0.001
GSH	2.99 (1.55–5.66)	3.86 (2.56–6.01)	0.74 (0.6 to 0.88)	<0.001

HA—hyaluronic acid; 95% CI—95% confidence interval; * Wilcoxon test; ^†^ Hodges–Lehman median difference.

**Table 3:** Absolute differences in measured parameters before and after therapy in both groups.

	Median (Interquartile Range) of Difference Before–After Therapy		Difference ^†^(95% CI)	*p* *
	Standard Therapy	Standard Therapy + HA		
TAC	−0.04 (−0.13–0)	−0.18 (−0.23 to −0.04)	−0.09 (−0.14 do −0.04)	<0.001
GR	−0.56 (−0.9 to −0.31)	−0.81 (−1.46 to −0.23)	−0.22 (−0.52 do 0)	0.05
UA	−34 (−59 to −12.75)	−72 (−92 to −34.5)	−29 (−43 to −15)	<0.001
GSH	−0.27 (−0.55 to −0.12)	−0.77 (−1.06 to −0.36)	−0.42 (−0.60 to −0.25)	<0.001

95% CI—95% confidence interval; * Mann–Whitney U-test; ^†^ Hodges–Lehmann median difference.

**Table 4:** Correlation between oxidative stress parameters and periodontal indices in patients with “standard” therapy.

Standard Therapy	Spearman Correlation Coefficient Rho (*p* Values)–before Therapy			
	TAC	GR	UA	GSH
Before therapy	oxidative stress parameters before therapy			
BoP	−0.927 (<0.001)	−0.939 (<0.001)	−0.832 (<0.001)	−0.92 (<0.001)
mean CAL	−0.505 (<0.001)	−0.511 (<0.001)	−0.392(<0.001)	−0.445 (<0.001)
PPD	−0.143 (0.32)	−0.057 (0.70)	−0.061 (0.68)	−0.038 (0.80)
After therapy	oxidative stress parameters before therapy			
BoP	−0.688 (<0.001)	−0.600(<0.001)	−0.627 (<0.001)	−0.609 (<0.001)
mean CAL	−0.261 (0.07)	−0.213 (0.14)	−0.056 (0.70)	−0.212 (0.14)
PPD	−0.079 (0.58)	0.001 (0.99)	−0.144 (0.32)	0.046 (0.75)
After therapy	oxidative stress parameters after therapy			
BoP	−0.656 (<0.001)	−0.588 (<0.001)	−0.501 (<0.001)	−0.591 (<0.001)
mean CAL	−0.219 (0.13)	−0.204 (0.16)	−0.221 (0.12)	−0.221 (0.12)
PPD	−0.084 (0.56)	−0.018 (0.90)	0.154 (0.29)	0.031 (0.83)

**Table 5:** Correlation between the parameters of oxidative stress and periodontal indices in patients with standard therapy + HA.

Standard Therapy + HA	Spearman Correlation Coefficient Rho (*p* Values)–before Therapy			
	TAC	GR	UA	GSH
Before therapy	oxidative stress indices before therapy			
BoP	−0.904 (<0.001)	−0.946 (<0.001)	−0.920 (<0.001)	−0.867 (<0.001)
Mean CAL	−0.572 (<0.001)	−0.627 (<0.001)	−0.535 (<0.001)	−0.543 (<0.001)
PPD	−0.318 (0.02)	−0.292 (0.04)	−0.268 (0.06)	−0.234 (0.10)
After therapy	oxidative stress indices before therapy			
BoP	−0.753 (<0.001)	−0.902 (<0.001)	−0.778 (<0.001)	−0.871 (<0.001)
Mean CAL	−0.469 (<0.001)	−0.633 (<0.001)	−0.519 (<0.001)	−0.548 (<0.001)
PPD	−0.303 (0.03)	−0.287 (0.04)	−0.268 (0.06)	−0.219 (0.13)
After therapy	oxidative stress indices after therapy			
BoP	−0.394 (<0.001)	−0.406 (<0.001)	−0.492 (<0.001)	−0.201 (0.16)
Mean CAL	−0.075 (0.61)	−0.097 (0.50)	−0.195 (0.17)	−0.248 (0.08)
PPD	−0.313 (0.03)	−0.38 (0.01)	−0.303 (0.03)	−0.3 (0.03)

## Data Availability

The data presented in this study are available on request from the corresponding author. The data are not publicly available due to ethical restrictions.
